# PEX11β and FIS1 cooperate in peroxisome division independently of mitochondrial fission factor

**DOI:** 10.1242/jcs.259924

**Published:** 2022-07-08

**Authors:** Tina A. Schrader, Ruth E. Carmichael, Markus Islinger, Joseph L. Costello, Christian Hacker, Nina A. Bonekamp, Jochen H. Weishaupt, Peter M. Andersen, Michael Schrader

**Affiliations:** 1 College of Life and Environmental Sciences, Biosciences, University of Exeter, Exeter EX4 4QD, UK; 2 Institute of Neuroanatomy, Mannheim Centre for Translational Neuroscience, Medical Faculty Mannheim, University of Heidelberg, 68167 Mannheim, Germany; 3 Division of Neurodegeneration, Department of Neurology, Mannheim Center for Translational Neurosciences, Medical Faculty Mannheim, Heidelberg University, 68167 Mannheim, Germany; 4 Department of Clinical Science, Neurosciences, Umeå University, Umeå SE-90185, Sweden

**Keywords:** Peroxisomes, Mitochondria, Organelle division, PEX11, FIS1, MFF

## Abstract

Peroxisome membrane dynamics and division are essential to adapt the peroxisomal compartment to cellular needs. The peroxisomal membrane protein PEX11β (also known as PEX11B) and the tail-anchored adaptor proteins FIS1 (mitochondrial fission protein 1) and MFF (mitochondrial fission factor), which recruit the fission GTPase DRP1 (dynamin-related protein 1, also known as DNML1) to both peroxisomes and mitochondria, are key factors of peroxisomal division. The current model suggests that MFF is essential for peroxisome division, whereas the role of FIS1 is unclear. Here, we reveal that PEX11β can promote peroxisome division in the absence of MFF in a DRP1- and FIS1-dependent manner. We also demonstrate that MFF permits peroxisome division independently of PEX11β and restores peroxisome morphology in PEX11β-deficient patient cells. Moreover, targeting of PEX11β to mitochondria induces mitochondrial division, indicating the potential for PEX11β to modulate mitochondrial dynamics. Our findings suggest the existence of an alternative, MFF-independent pathway in peroxisome division and report a function for FIS1 in the division of peroxisomes.

This article has an associated First Person interview with the first authors of the paper.

## Abstract (precis)

**Summary:** We show that PEX11β can promote peroxisome division in the absence of MFF in a DRP1- and FIS1-dependent manner, suggesting that an alternative, MFF-independent pathway in peroxisome division exists.

## INTRODUCTION

Organelle membrane dynamics and division are important processes for the modulation and regulation of organelle abundance within eukaryotic cells, which are required to adapt to metabolic changes, cell cycle progression and autophagy (Mast et al., 2020; Yapa et al., 2021). Defects in organelle division have been linked to severe disorders with neurological abnormalities (Chan, 2020; Waterham et al., 2007).

Peroxisomes are single-membrane-bound organelles with important roles in cellular lipid and hydrogen peroxide metabolism. They contribute to multiple metabolic processes, including fatty acid β-oxidation and the synthesis of ether phospholipids (such as myelin sheath lipids) (Wanders and Waterham, 2006). Peroxisomes can rapidly proliferate and increase their numbers via growth (elongation) and division, for example, upon stimulation with fatty acids or certain chemicals (peroxisome proliferators) (Schrader et al., 2016). Peroxisomes are not only metabolically linked to mitochondria but also share components of their division machinery (Schrader et al., 2015). These include the tail-anchored adaptor proteins FIS1 and MFF, which are dually targeted to both peroxisomes and mitochondria, where they recruit the fission GTPase DRP1 (also known as DNML1) to the organelle membrane (Costello et al., 2017b, 2018; Gandre-Babbe and van der Bliek, 2008; Koch et al., 2005). In contrast to mitochondria, peroxisomes do not fuse (Bonekamp et al., 2012; Huybrechts et al., 2009); elongation is instead mediated by the peroxisome-specific membrane protein and biogenesis factor PEX11β (also known as PEX11B) (Schrader et al., 1998), with lipids for membrane expansion likely provided by the endoplasmic reticulum (ER) via peroxisome–ER contacts (Costello et al., 2017a; Hua et al., 2017; Islinger et al., 2020). PEX11β is a key factor in the regulation of peroxisome abundance in mammals (Schrader et al., 2016). It functions as a membrane-remodelling protein, using N-terminal amphipathic helices that interact with membrane lipids to deform and elongate the peroxisome membrane prior to fission (Delille et al., 2010; Koch et al., 2003; Opaliński et al., 2011; Yoshida et al., 2015). Furthermore, PEX11β interacts with FIS1 and MFF to aid assembly of the fission machinery (Itoyama et al., 2013; Kobayashi et al., 2007; Koch and Brocard, 2012; Koch et al., 2005) and stimulates the GTPase activity of DRP1 (Williams et al., 2015).

In the current model of DRP1-mediated organelle division, MFF is considered the major receptor for DRP1 on peroxisomes and mitochondria in mammals (Otera et al., 2010). However, how the division components FIS1, MFF, PEX11β and DRP1 cooperate to mediate peroxisome division is still unclear. Loss of MFF function results in highly elongated peroxisomes and mitochondria in cultured mammalian cells and skin fibroblasts from patients with MFF deficiency (Gandre-Babbe and van der Bliek, 2008; Otera et al., 2010; Passmore et al., 2020). MFF deficiency presents as developmental delay, peripheral neuropathy, optic atrophy and Leigh-like encephalopathy (Koch et al., 2016; Nasca et al., 2018; Shamseldin et al., 2012). The role of FIS1 in division is still unclear and controversial, because its loss does not cause a pronounced elongation of peroxisomes or mitochondria (Ihenacho et al., 2021). Additional functions for mitochondrial FIS1 in mitophagy and apoptosis have been reported (Alirol et al., 2006; Iwasawa et al., 2011). Whereas patients with FIS1 deficiency have not yet been described, patients with a loss of PEX11β function have been identified (Ebberink et al., 2012; Taylor et al., 2017; Tian et al., 2020) and present with short stature, eye problems, progressive hearing loss and neurological defects. The metabolic functions of peroxisomes and mitochondria are either not or only moderately affected in DRP1-, MFF- or PEX11β-deficient patients (Passmore et al., 2020; Waterham et al., 2007), suggesting that the symptoms relate to decreased organelle plasticity.

Here, we reveal that PEX11β can promote peroxisome division in the absence of MFF in a DRP1-dependent manner. We show that PEX11β requires FIS1 to induce peroxisome division in the absence of MFF and that MFF can restore peroxisome division in PEX11β-deficient patient fibroblasts. Interestingly, targeting of PEX11β to mitochondria induces mitochondrial division, revealing the potential for PEX11β to modulate mitochondrial dynamics. Our findings indicate that MFF and FIS1 can act independently in peroxisome division, and suggest the existence of an alternative MFF-independent pathway in peroxisome division that depends on PEX11β and FIS1.

## RESULTS

### PEX11β induces peroxisome division in MFF-deficient fibroblasts in a DRP1-dependent manner

**Fig. 1.**
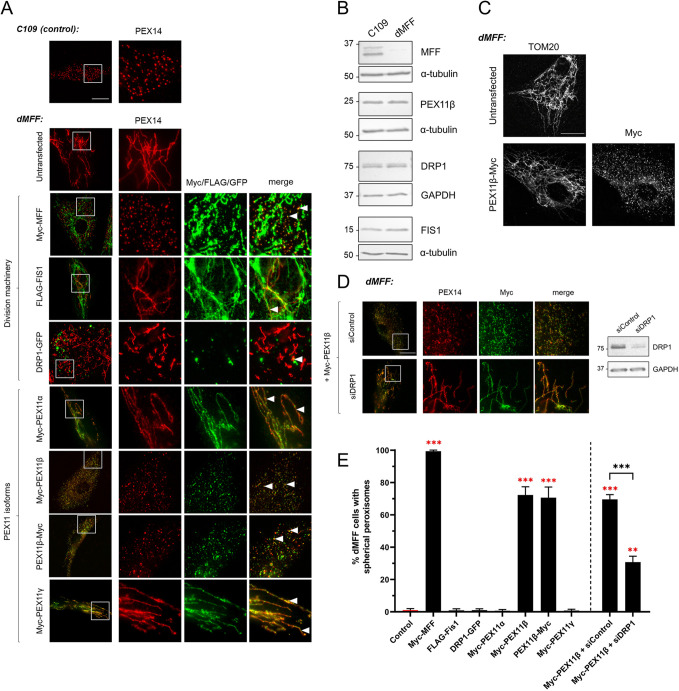
**Expression of PEX11β in dMFF fibroblasts induces peroxisome division in a DRP1-dependent manner.** (A) Representative images of peroxisome morphology in control fibroblasts (C109) and in MFF-deficient fibroblasts (dMFF) that were either untransfected or transfected with Myc–MFF, FLAG–FIS1, DRP1–GFP, Myc–PEX11α, Myc–PEX11β, PEX11β–Myc or Myc–PEX11γ. Arrowheads highlight colocalisation. (B) Immunoblots of cell lysates from control (C109) and MFF-deficient fibroblasts (dMFF) using anti-MFF, anti-FIS1, anti-DRP1 and anti-PEX11β antibodies. Loading controls used were α-tubulin and GAPDH. Blots shown are representative of three experiments. (C) Representative images of mitochondrial morphology in control untransfected or PEX11β–Myc-transfected dMFF cells. Images represent three experiments. (D) Left: representative images of peroxisome morphology in dMFF cells after expression of PEX11β (Myc–PEX11β) and either silencing of DRP1 (siDRP1) or treatment with control siRNA (siControl). Right: silencing was confirmed by immunoblotting with an anti-DRP1 antibody. GAPDH served as a loading control. Fixed cells in A, C and D were labelled with anti-Myc or anti-FLAG antibodies, as appropriate, and with anti-PEX14 (peroxisomal marker; A,D) or anti-TOM20 (mitochondrial marker; C) antibodies. Boxes in the left-hand merge images in A and D indicate regions shown in the higher magnification images to the right. Scale bars: 20 µm. Immunoblot molecular mass markers shown in B and D indicate kDa. (E) Quantification of peroxisome morphology (for experiments as described in A and D; *c*ontrol, untransfected dMFF cells). Data are presented as mean±s.d. of at least three independent experiments, with *n*=500–1300 cells in total. **P<0.01; ***P<0.001 (Brown–Forsythe and Welch one-way ANOVA with Dunnett's multiple comparisons test).

To assess the contributions and interplay of different components of the division machinery, we first investigated whether overexpressing peroxisome division factors can rescue peroxisome fission in MFF-deficient human fibroblasts (dMFF) as a cellular model. Peroxisomes in dMFF cells are highly elongated tubular structures positive for the peroxisomal membrane protein PEX14, and they can be clearly distinguished from the small, spherical peroxisomes observed in control fibroblasts (C109) (Fig. 1A). This hyper-elongated morphology is caused by compromised peroxisome division whilst membrane elongation (growth) still proceeds (Costello et al., 2017a; Passmore et al., 2020; Shamseldin et al., 2012). Occasionally, dMFF cells with shorter and spherical peroxisomes are observed, indicating that division can proceed in rare cases, despite the absence of MFF. Notably, other proteins involved in peroxisome division (PEX11β, FIS1 and DRP1) were expressed at normal levels in these cells (Fig. 1B), suggesting that the division defect is specifically due to loss of MFF. Accordingly, re-expression of MFF in these cells was sufficient to induce division of the hyper-elongated peroxisomes into numerous spherical forms (Fig. 1A,E). Overexpression of FIS1 or DRP1 in dMFF cells could not restore peroxisome division. Surprisingly, however, overexpression of PEX11β was sufficient to induce division of the hyper-elongated peroxisomes. The spherical peroxisomes generated by PEX11β expression were positive for other peroxisomal marker proteins such as ACBD5 and PMP70 (also known as ABCD3), which are involved in fatty acid uptake, indicating that they represent mature peroxisomes (Fig. S1). Mitochondria did not divide when PEX11β was expressed in dMFF cells (Fig. 1C). The PEX11α (PEX11A) and PEX11γ (PEX11G) isoforms did not restore the spherical peroxisome morphology, pointing to different functions at peroxisomes (Azadi et al., 2020) (Fig. 1A,E). This indicates that in our experimental setup, PEX11β can drive peroxisome division in the absence of MFF and, importantly, suggests that MFF can be dispensable for peroxisome fission.

PEX11β has no intrinsic division activity, and peroxisome division depends on DRP1 (Koch et al., 2004). To investigate whether PEX11β-mediated peroxisome division in dMFF cells is DRP1 dependent, we silenced DRP1 by transfecting the cells with a previously characterised, potent DRP1 siRNA (Koch et al., 2004) prior to expression of PEX11β. PEX11β-driven peroxisome division was significantly reduced after silencing of DRP1, demonstrating that this process depends on DRP1 (Fig. 1D,E).

### The conserved C-terminal tail of PEX11β is essential to drive peroxisome division

PEX11β is involved in several steps of peroxisomal growth and division: it remodels and elongates the peroxisome membrane prior to fission, assembles components of the division machinery and stimulates the GTPase activity of DRP1 (Schrader et al., 2016). It has therefore been difficult to delineate the impact of PEX11β mutations on the final division step, as mutations that inhibit the initial step of membrane elongation, a pre-requisite of peroxisome division, thus also block fission. MFF-deficient fibroblasts provide an excellent model to investigate the impact of PEX11β mutations on the final division step, as peroxisomes are already elongated in dMFF cells.

**Fig. 2.**
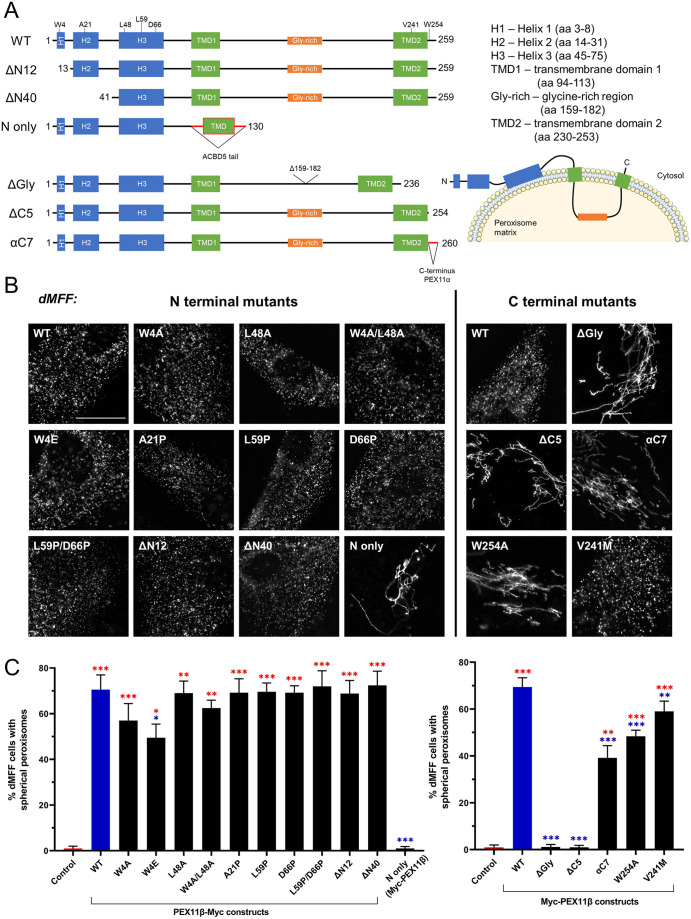
**Ability of PEX11β mutants to divide peroxisomes in dMFF fibroblasts.** (A) Schematic of wild-type (WT) PEX11β and the PEX11β mutants used (aa, amino acids), with mutated amino acid residues indicated, and a cartoon showing topology of human PEX11β in the peroxisome membrane. (B) Representative images of peroxisome morphology in MFF-deficient fibroblasts (dMFF) transfected with PEX11β–Myc WT or N-terminal mutants, or with Myc–PEX11β WT or C-terminal mutants. Anti-Myc labelling of fixed cells is shown. Scale bar: 20 µm. (C) Quantification of peroxisome morphology for dMFF cells expressing the indicated PEX11β constructs (control, untransfected dMFF cells). Data are presented as mean±s.d. of at least three experiments, with *n*=400–1300 cells in total. *P<0.05; **P<0.01; ***P<0.001 (Brown–Forsythe and Welch one-way ANOVA with Dunnett's multiple comparisons test).

To probe the molecular mechanism underlying MFF-independent, PEX11β-driven peroxisome division, we expressed PEX11β mutants in dMFF cells and assayed their ability to induce fission of the hyper-elongated peroxisomes (Fig. 2; Table 1). We first investigated the impact of mutations within the N terminus of PEX11β, which contains amphipathic helices for lipid interaction and PEX11β self-interaction, as well as residues important for the regulation of DRP1 GTPase activity (Fig. 2A,B; Table 1). The conserved residues Trp4 (helix 1) and Leu48 have been implicated in PEX11β–DRP1 interaction and regulation of DRP1 GTPase activity. Expression of PEX11β^W4A^, but not PEX11β^L48A^, has been shown to compromise peroxisome fission in COS-7 cells (Williams et al., 2015). We therefore expressed PEX11β^W4A^, PEX11β^W4E^, PEX11β^L48A^ and PEX11β^W4A-L48A^ constructs in dMFF cells and analysed peroxisome morphology using immunofluorescence (Fig. 2B,C). Of these, PEX11β^W4E^ moderately reduced peroxisome division compared to that observed when wild-type PEX11β (PEX11β^WT^) was expressed, likely due to its stronger interference with the amphipathic properties of helix 2, supporting a role for Trp4 in the regulation of peroxisome division. Next, we examined the function of the N-terminal amphipathic helices (helix 2 and 3) in peroxisome division in dMFF cells. PEX11β^A21P^ (helix 2), PEX11β^D66P^ (helix 3), PEX11β^L59P^ (helix 3) and PEX11β^D66P-L59P^ (helix 3) mutants contain at least one helix-breaking proline within helix 2 or 3. Interestingly, none of these mutants inhibited PEX11β-dependent peroxisome division in dMFF cells, indicating that, while these amphipathic helices are reported to have a major role in membrane elongation (Opaliński et al., 2011; Su et al., 2018), they are not required for division (Table 1). We then expressed the N-terminal truncations PEX11β^ΔN12^ and PEX11β^ΔN40^ in dMFF cells. As shown in Fig. 2 and previously (Bonekamp et al., 2013a), these were properly targeted to peroxisomes, as the binding site for PEX19, the peroxisome-targeting receptor for membrane proteins, is located in the C-terminal region (Sacksteder et al., 2000). Surprisingly, both N-terminally truncated PEX11β proteins were able to induce peroxisome division similar to PEX11β^WT^, indicating that the N-terminal 40 amino acids are dispensable for peroxisome division in dMFF cells and implying that other regions within PEX11β contribute to DRP1 interaction and regulation (Table 1). This appears to be in contrast to observations made with PEX11β^W4E^, which revealed moderately reduced peroxisome division (Fig. 2B,C), suggesting that this mutation might have a dominant-negative effect. PEX11β^ΔN40^ and PEX11β^A21P^ have also been shown to impair self-interaction of PEX11β, which is important for PEX11β-mediated membrane elongation of peroxisomes (Bonekamp et al., 2013a; Itoyama et al., 2012; Su et al., 2018). As both mutants permit peroxisome division, self-interaction of PEX11β does not appear to be essential for final peroxisome scission. Expression of the PEX11β N terminus [fused to the transmembrane domain (TMD) and tail region of ACBD5, a peroxisomal tail-anchored membrane protein] did not promote peroxisome division in dMFF cells, indicating that the N-terminal region is not sufficient to induce membrane fission (Table 1).

**Table 1.**
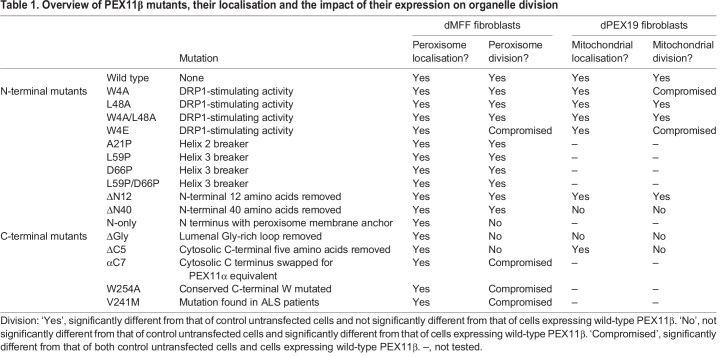
Overview of PEX11β mutants, their localisation and the impact of their expression on organelle division

We then investigated alterations of the PEX11β C-terminal region. Human PEX11β contains a glycine-rich region (amino acids 159–182) that is located between the two TMDs and is exposed to the peroxisome matrix (Fig. 2A) (Bonekamp et al., 2013a). Interestingly, this glycine-rich region is conserved in mammals, but is absent in PEX11α and PEX11γ (Fig. S2). A version of PEX11β with the glycine-rich region deleted (PEX11β^ΔGly^) was properly targeted to peroxisomes in dMFF cells (Fig. 2B; Fig. S3; Table 1), but surprisingly, was unable to induce peroxisome division. Similarly, a version with a five-amino-acid truncation of the C terminus (PEX11β^ΔC5^) was targeted to peroxisomes (Fig. 2B; Fig. S3; Table 1) (Delille et al., 2010), but did not induce peroxisome division in dMFF cells. A comparison of PEX11β protein sequences from different species revealed a high conservation of the C-terminal tail, in particular Trp254, which is specific for PEX11β and is absent in PEX11α and PEX11γ (Fig. S2). Exchanging the C-terminal tail of PEX11β for the cytosolic tail of PEX11α (C-terminal seven amino acids, PEX11β^αC7^), or mutating the conserved Trp254 residue (PEX11β^W254A^), significantly reduced, but did not abolish, the ability of PEX11β to divide the elongated peroxisomes in dMFF cells (Fig. 2B,C). Our findings indicate that the C-terminal region, and not the N terminus, of PEX11β is essential for peroxisome division in dMFF cells (Table 1).

We also analysed the effect of a mutation in the C-terminal TMD (PEX11β^V241M^) (Fig. 2B,C; Fig. S3; Table 1). The Val241Met variant has been found in patients suffering from amyotrophic lateral sclerosis (ALS) from Germany (one familial patient, one sporadic patient) and Finland (one familial patient with ALS and dementia) by whole-exome sequencing. For further details regarding these patients please see the Materials and Methods. Peroxisome defects have previously been described in neurodegenerative disorders including ALS, a fatal disease that is characterised by adult-onset progressive loss of upper and lower motor neurons (Andrés-Benito et al., 2021). When expressed in dMFF cells, PEX11β^V241M^ induced peroxisome division, but less efficiently than PEX11β^WT^. No other genetic variants of significance for ALS pathogenesis were observed in these patients, suggesting that the PEX11β^V241M^ may possibly be contributing to the phenotype, but this requires further investigation. This experiment demonstrates that MFF-deficient human fibroblasts could represent a suitable model to analyse the impact of PEX11β mutations on peroxisome fission in a neurodegenerative disease.

### MFF expression in PEX11β-deficient cells induces peroxisome division

**Fig. 3.**
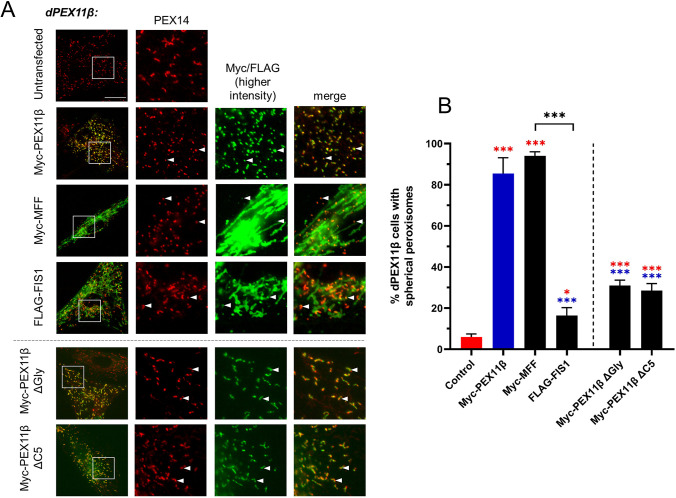
**Expression of MFF in PEX11β-deficient fibroblasts induces peroxisome division.** (A) Peroxisome morphology in PEX11β-deficient fibroblasts (dPEX11β) that were either untransfected or transfected with Myc–PEX11β, Myc–MFF, FLAG–FIS1, Myc–PEX11β^ΔC5^ or Myc–PEX11β^ΔGly^. Fixed cells were labelled with anti-Myc or anti-FLAG antibodies, as appropriate, and with an anti-PEX14 antibody. Boxes in the left-hand images indicate regions shown in magnified images to the right. Arrowheads highlight colocalisation. Scale bar: 20 µm. (B) Quantification of peroxisome morphology in control untransfected dPEX11β cells and dPEX11β cells expressing the indicated constructs. Data are presented as mean±s.d. from at least three independent experiments, with *n*=300–500 cells in total. ***P<0.001 (ordinary one-way ANOVA with Tukey's multiple comparisons test).

Previous studies have suggested that the glycine-rich region and the five-amino-acid C-terminal cytoplasmic tail of PEX11β are dispensable for peroxisome division (Bonekamp et al., 2013a; Delille et al., 2010). This contrasts with our observations in dMFF cells and may be explained by the existence of independent peroxisome division pathways in mammalian cells. We therefore hypothesised that MFF and PEX11β may be able to act independently in peroxisome fission. To test this, we overexpressed MFF in PEX11β-deficient fibroblasts (dPEX11β). Under basal conditions, dPEX11β fibroblasts displayed slightly elongated (rod-shaped) peroxisomes (Fig. 3), consistent with the role of PEX11β in peroxisome division (Azadi et al., 2020). PEX11γ can partially complement the dPEX11β phenotype (Ebberink et al., 2012), so it is likely that the minor elongation in the absence of PEX11β is due to the action of PEX11γ (Koch et al., 2010). Remarkably, overexpression of MFF in dPEX11β cells was sufficient to induce division of the rod-shaped peroxisomes to the same extent as re-introduction of PEX11β (Fig. 3). Expression of FIS1, however, did not restore peroxisome division in dPEX11β cells. In addition, expression of the C-terminal mutants PEX11β^ΔGly^ and PEX11β^ΔC5^ did not fully restore peroxisome division and a spherical peroxisome phenotype in the majority of cells. We conclude from these observations that MFF can act independently of PEX11β in peroxisome division. In line with this, it has been shown that MFF can stimulate DRP1 GTPase activity itself, so may compensate for that aspect of PEX11β activity (Clinton et al., 2016; Losón et al., 2013; Macdonald et al., 2016; Osellame et al., 2016).

### PEX11β-induced division of peroxisomes depends on FIS1

**Fig. 4.**
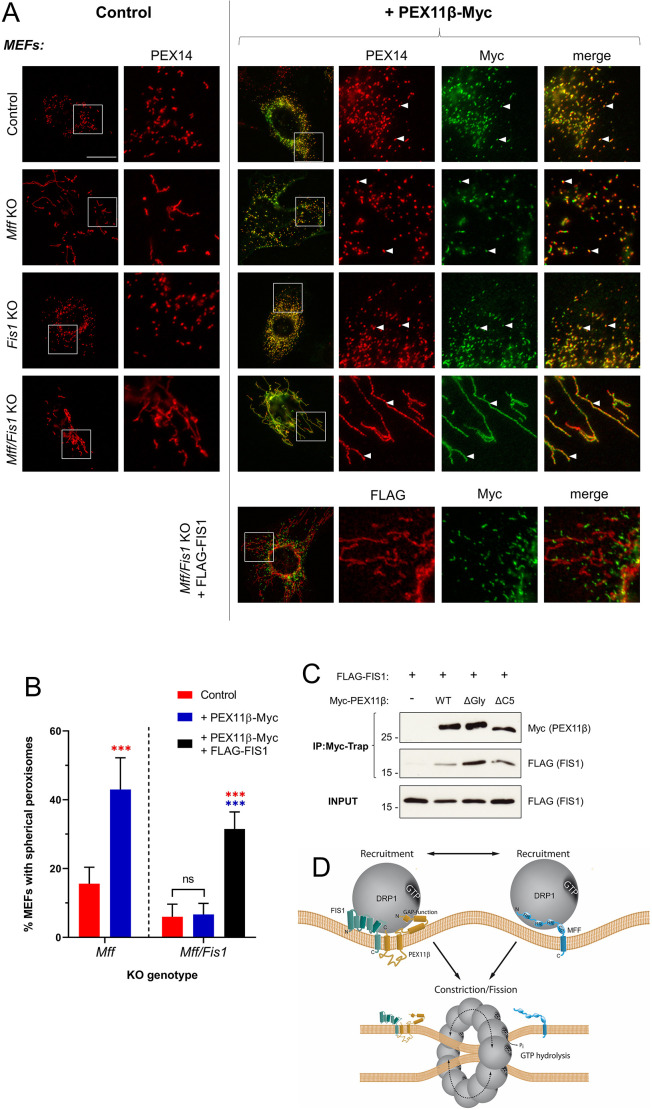
**PEX11β-induced division of peroxisomes requires FIS1.** (A) Peroxisome morphology in MEF control, *Mff* single KO, *Fis1* single KO and *Mff Fis1* double KO (*Mff/Fis1* KO) cells that were either untransfected (control) or transfected with PEX11β–Myc or with PEX11β–Myc and FLAG–FIS1. Fixed cells were labelled with anti-Myc and anti-PEX14 or anti-FLAG antibodies, as indicated. Boxes in the left-hand images indicate regions shown in magnified images to the right. Arrowheads highlight colocalisation. Scale bars: 20 µm. Images are representative of three experiments. (B) Quantification of peroxisome morphology in *Mff* KO and *Mff/Fis1* KO MEFs transfected with the indicated constructs. Data are presented as mean±s.d. from at least three independent experiments, with *n*=500–1200 cells in total. ***P<0.001; ns, not significant (two-way ANOVA with Sidak's multiple comparisons test). (C) Immunoprecipitation (IP) of FLAG–FIS1 and Myc–PEX11β (wild-type PEX11β, WT; PEX11β^ΔGly^, ΔGly; PEX11β^ΔC5^, ΔC5) after co-expression in COS-7 cells. Samples were immunoprecipitated (Myc-Trap) and immunoblotted using anti-Myc and anti-FLAG antibodies. Immunoblot molecular mass markers are shown in kDa. Input blot represents 1% of the total lysate. Blots shown are representative of three experiments. (D) Working model of PEX11β–FIS1-dependent and MFF-dependent peroxisome division. Details are based on this study and published data (Dohm et al., 2004; Kobayashi et al., 2007; Opaliński et al., 2011; Bonekamp et al., 2013a; Koch and Brocard, 2012; Williams et al., 2015). Please note that this is a hypothetical model. CC, coiled-coil domain; GAP, GTPase-activating protein; P_i_, inorganic phosphate; RR, repetitive region.

Our data suggest the existence of parallel pathways of peroxisome division that depend on either MFF or PEX11β. PEX11β reportedly interacts with FIS1 at the peroxisome membrane (Kobayashi et al., 2007). To probe the role of FIS1 in MFF-independent, PEX11β-dependent peroxisome fission, we overexpressed PEX11β in *Mff* and *Fis1* knockout (KO) mouse embryonic fibroblasts (MEFs), and assayed its ability to induce peroxisome division. As in the dMFF cells, *Mff* KO MEFs displayed elongated peroxisomes that were divided into spherical forms upon PEX11β overexpression (Fig. 4A,B). Peroxisomes were spherical under basal conditions in *Fis1* KO MEFs, likely because MFF-dependent peroxisome fission was still active. In line with this, PEX11β expression in *Fis1* KO cells was able to induce peroxisome multiplication. MEFs lacking both *Mff* and *Fis1* (double KO) exhibited elongated peroxisomes, and this elongation was often more pronounced than that observed in the *Mff* single KO. Importantly, peroxisome division was not induced by PEX11β overexpression in the double KO cells (Fig. 4A,B), suggesting a key role for FIS1 in PEX11β-dependent peroxisome division. Indeed, co-expression of FIS1 and PEX11β in double KO cells significantly restored peroxisome division (Fig. 4). Our findings also suggest that MFF and FIS1 can act independently of each other in peroxisome division.

The interaction of FIS1 and PEX11β is thought to be mediated by the C terminus of PEX11β (Kobayashi et al., 2007). As alterations of the PEX11β C terminus were found to block the division of peroxisomes in dMFF cells, we reasoned that PEX11β^ΔC5^ and PEX11β^ΔGly^ might show a reduced interaction with FIS1. However, co-expression of these mutants with FIS1 in COS-7 cells and subsequent immunoprecipitation did not reveal an obvious reduction in FIS1 binding compared to that exhibited by PEX11β^WT^ (Fig. 4C). We therefore speculate that the C-terminal alterations to PEX11β impact on the formation of a functional complex, which might then be unable to trigger DRP1-mediated peroxisome division as a result. Loss of the glycine-rich region, which likely provides flexibility to PEX11β, as well as the loss of the C-terminal cytoplasmic tail, might alter the conformation or properties of a PEX11β–FIS1–DRP1 division complex and render it non-functional. Interaction between MFF and PEX11β has also been described (Itoyama et al., 2013; Koch and Brocard, 2012), so we cannot rule out that PEX11β also interacts with MFF to regulate peroxisome division under conditions where both MFF and FIS1 are present. In summary, our findings show that PEX11β requires FIS1 and DRP1 to promote peroxisome division in the absence of MFF (Fig. 4D).

### PEX11β induces mitochondrial division when routed to mitochondria

**Fig. 5.**
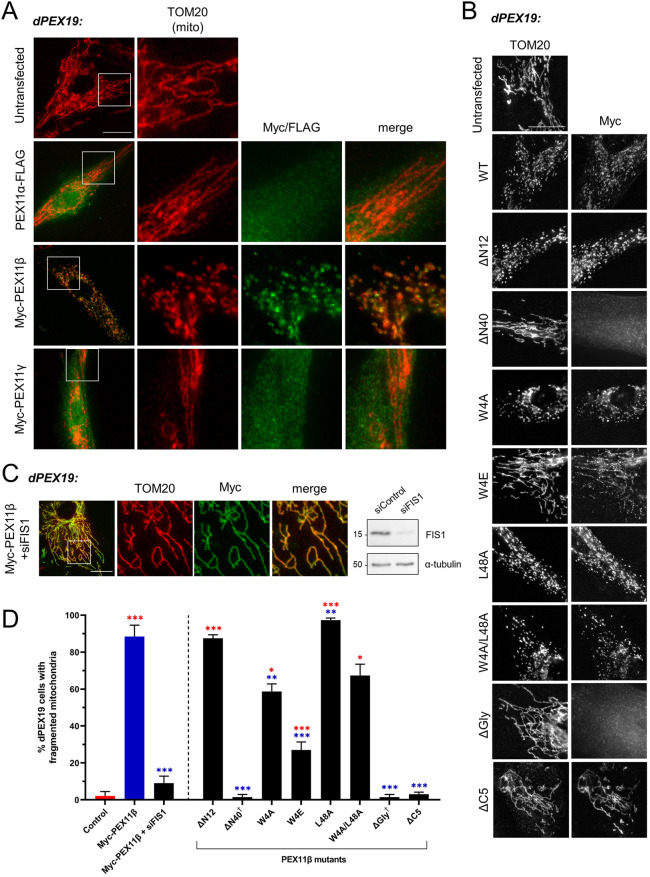
**PEX11β targets to mitochondria and induces mitochondrial division in dPEX19 cells.** (A) Representative images of mitochondrial morphology in PEX19-deficient cells (dPEX19) lacking peroxisomes transfected with PEX11α–FLAG, Myc–PEX11β, and Myc–PEX11γ. (B) Representative images of mitochondrial morphology in dPEX19 cells transfected with the indicated PEX11β–Myc N-terminal mutants or Myc–PEX11β C-terminal mutants. WT, Myc–PEX11β. (C) Representative images of mitochondrial morphology in dPEX19 cells transfected with Myc–PEX11β after silencing of FIS1 (siFIS1). Silencing was confirmed by immunoblotting with anti-FIS1 antibodies (siControl, control siRNA). Tubulin served as a loading control. Immunoblot molecular mass markers are shown in kDa. Fixed cells in A–C were labelled with anti-Myc or anti-FLAG antibodies, as appropriate, and with an anti-TOM20 (mitochondrial marker) antibody. Boxes in left-hand images of A and C indicate regions shown in the magnified images to the right. Scale bars: 20 µm. (D) Quantification of mitochondrial morphology in experiments as described for A–C. Control, untransfected dPEX19 cells. Data are presented as mean±s.d. from at least three independent experiments, with *n*=300–1500 cells in total. † indicates mutants not targeted to mitochondria. *P<0.05; **P<0.01; ***P<0.001 (Brown–Forsythe and Welch one-way ANOVA with Dunnett's multiple comparisons test).

**Fig. 6.**
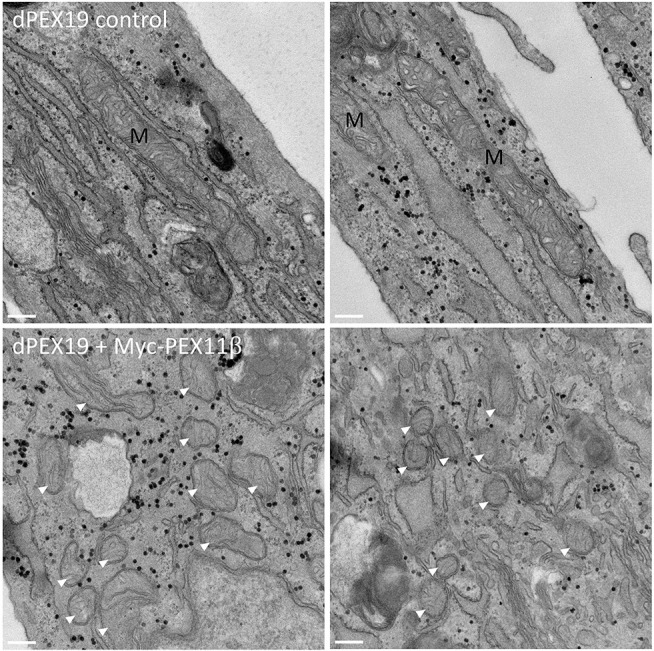
**Spherical mitochondria, which form through PEX11β-driven mitochondrial division in dPEX19 cells, maintain an intact morphology.** Representative electron micrographs of PEX19-deficient fibroblasts (dPEX19 control, upper panels) and dPEX19 cells transfected with Myc–PEX11β (lower panels). Note the elongated morphology of mitochondria (M) in control cells, whereas mitochondria are spherical in Myc–PEX11β-expressing cells. The spherical mitochondria (arrowheads) appear intact, with cristae and association with the ER. Scale bars: 200 nm. Images are representative of randomly sampled cells from two experiments per experimental condition.

Mistargeting of peroxisomal membrane proteins (including PEX11β) to mitochondria has been described in mammalian cells under conditions where peroxisomes are lost and peroxisome membrane insertion is compromised (Costello et al., 2017b; Sacksteder et al., 2000). PEX19-deficient fibroblasts (dPEX19) lack functional peroxisomes, because PEX19 is required for peroxisome biogenesis. When PEX11β was expressed in dPEX19 cells, it was routed to mitochondria due to the absence of peroxisomes (Fig. 5). PEX11α and PEX11γ, on the other hand, did not localise at mitochondria in dPEX19 fibroblasts (Fig. 5). Interestingly, while staining of the mitochondrial marker TOM20 (also known as TOMM20) showed that dPEX19 cells almost exclusively contained elongated mitochondria under basal conditions, overexpression of PEX11β (but not PEX11α or PEX11γ) caused the mitochondria to divide into spherical forms. Ultrastructural analysis of dPEX19 fibroblasts expressing PEX11β confirmed the presence of intact spherical mitochondria with cristae, suggesting bona fide division (Fig. 6). Differential permeabilisation after expression of N- and C-terminally Myc-tagged variants of PEX11β and subsequent immunofluorescence with an anti-Myc antibody or an anti-PEX11β antibody (directed against an epitope in the internal loop; see Fig. 2A) indicated that PEX11β retained its native topology within the mitochondrial outer membrane with the N- and C-termini exposed to the cytosol (Fig. S4). In addition, mitochondrial PEX11β could be extracted from the membrane by post-fixation Triton X-100 treatment, as previously reported for peroxisomal PEX11β (Schrader et al., 2012), suggesting that its properties within the membrane are retained (Fig. S4).

Expression of the N-terminal variants PEX11β^W4A^ and PEX11β^W4E^ resulted in reduced division of mitochondria in dPEX19 cells relative to that observed following expression of PEX11β^WT^, whereas PEX11β^L48A^ and PEX11β^W4A-L48A^ did not compromise PEX11β-induced mitochondrial division (Fig. 5; Table 1). PEX11β^W4E^ expression also resulted in a reduced division of peroxisomes in dMFF cells (Fig. 2B,C; Table 1), underlining the importance of Trp4 for PEX11β-mediated division. PEX11β^A21P^ (helix 2) and PEX11β^ΔN12^ were both able to induce mitochondrial division similar to PEX11β^WT^. However, PEX11β^ΔN40^ was no longer targeted to mitochondria (Fig. 5B,D; Table 1), indicating that the N-terminal region comprising amino acids 12–40 is required for mitochondrial targeting. Adding a large yellow fluorescent protein (YFP) tag to the N terminus of PEX11β also abolished mitochondrial localisation, whereas C-terminal tagging did not interfere with mitochondrial localisation (Fig. S4). PEX11β^ΔGly^ was also not targeted to mitochondria in dPEX19 cells. However, the C-terminal variant PEX11β^ΔC5^ was targeted to mitochondria, but could not induce mitochondrial division (Fig. 5B,D; Table 1). These observations confirm the importance of the C-terminal tail for PEX11β-mediated division. As mitochondria and peroxisomes share key components of their division machinery, we hypothesise that PEX11β, in the mitochondrial outer membrane, interacts with FIS1 and DRP1 to drive mitochondrial division. In support of this, silencing of FIS1 in dPEX19 cells prior to PEX11β expression reduced mitochondrial division (Fig. 5C,D). MFF has already been shown to promote mitochondrial division independently of FIS1 (Clinton et al., 2016; Losón et al., 2013; Macdonald et al., 2016; Osellame et al., 2016). As PEX11β reportedly interacts with MFF, it is possible that this interaction can also promote mitochondrial division. There is also evidence for an overlap between PEX11β and mitochondrial function, as PEX11β is co-regulated with mitochondrial proteins (Kustatscher et al., 2019).

## DISCUSSION

We show here that PEX11β is able to drive peroxisome division in the absence of MFF. Furthermore, our data support a role for FIS1 in the MFF-independent peroxisome division induced by PEX11β. As our experiments are based on overexpression, the physiological importance of this pathway remains to be elucidated. However, to estimate what level of PEX11β is required to induce peroxisome division in MFF deficient cells, we processed the cells at early time points (6, 12 and 24 h after transfection) for immunofluorescence and immunoblotting (Fig. S5). Previous studies have revealed that the use of electroporation or microporation allows detection of PEX11β within hours after transfection (Schrader et al., 1998). Quantification of peroxisome morphology indicated that ∼30% of the PEX11β-expressing cells have spherical peroxisomes after 12 h, and ∼70% after 24 h. The corresponding immunoblots, which show endogenous as well as overexpressed PEX11β, indicate that the level of PEX11β might need to increase ∼2.5 fold to promote division, which could be feasible under physiological conditions.

Overall, our data indicate that alternative, independent pathways may exist for the division of peroxisomes in mammalian cells (Fig. 4D). Several lines of evidence from our experimental setup support this: (1) PEX11β promotes peroxisome division in the absence of MFF, requiring FIS1 and DRP1; (2) MFF can permit division of peroxisomes independently of PEX11β; and (3) PEX11β is unable to promote peroxisome division in the absence of both MFF and FIS1. The role of FIS1 in peroxisome (and mitochondrial) division has been unclear (Ihenacho et al., 2021). We demonstrate here that FIS1 needs PEX11β as a ‘co-factor’ for peroxisome division. PEX11β and FIS1 likely cooperate to form a functional division complex, which depends on the C-terminal region of PEX11β, whereas MFF can divide peroxisomes independently of PEX11β and FIS1 (Fig. 4D). This might need to be considered when interpreting previous findings on peroxisome division that are based on manipulation of MFF, FIS1 or PEX11β. MFF is only found in metazoans, indicating that the PEX11–FIS1 pathway presumably developed earlier in evolution. Indeed, deletion of FIS1 in fungi alters peroxisome dynamics (Garrido-Bazán et al., 2020; Navarro-Espíndola et al., 2020). Given that a close interrelationship between mitochondria and peroxisomes has evolved in animals (Schrader et al., 2015), we speculate that the acquisition of an MFF-dependent additional pathway has improved coordinated division of mitochondria and peroxisomes. PEX11β is a key factor in peroxisome proliferation, and together with FIS1, it might allow regulation of peroxisome division more independently of mitochondrial division. It is possible that further fine-tuning of peroxisome division is mediated by an interaction between PEX11β and MFF.

Our findings might also be important for understanding of the role of FIS1 at mitochondria. It is currently assumed that FIS1 is involved in specialised fission (e.g. in stress conditions), whereas MFF is the main DRP1 adaptor for fission. In line with this, a role for MFF in midzone fission of mitochondria for biogenesis and a role for FIS1 in peripheral fission for mitochondrial degradation have been reported (Kleele et al., 2021). A more specialised role for FIS1 might also apply to peroxisomes, but additional mitochondrial ‘co-factors’ could also be required to trigger FIS1-mediated mitochondrial fission. Interestingly, FIS1 recruits TBC1D15 and TBC1D17 to mitochondria, which – like PEX11β – have GTPase stimulating activity, in this case towards Rab7 and Rab8 proteins in mitophagy (Yamano et al., 2014). It remains to be elucidated whether FIS1 also contributes to pexophagy.

As mitochondria in dPEX19 fibroblasts are usually elongated, it is unlikely that PEX11β is sufficiently concentrated at mitochondria to trigger division under those basal conditions. However, this might be different in other cell types of PEX19-deficient patients. Furthermore, environmental changes and signalling events, which induce peroxisome proliferation (e.g. metabolic alterations, stress conditions) can potentially promote expression of PEX11β, resulting in mistargeting and division of mitochondria when peroxisomal targeting is impaired (Azadi et al., 2020; Delmaghani et al., 2015). Currently, there is little information available on the regulation of PEX11β expression, which complicates more physiological studies. Although mitochondrial alterations and mistargeting of peroxisomal proteins to mitochondria have been observed in peroxisome biogenesis disorders (Nuebel et al., 2021; Peeters et al., 2015), it remains to be established whether PEX11β mistargeting contributes to the pathophysiology of PEX19-deficiency and related disorders. However, our findings highlight the potential for PEX11β to modulate mitochondrial dynamics.

An intriguing finding of our study is the observation that expression of MFF in dPEX11β fibroblasts, or PEX11β in dMFF patient cells, can restore peroxisome division and, thus, the peroxisomal phenotype. Our observations suggest that modulation of MFF or PEX11β protein levels might represent a therapeutic option to overcome defects in peroxisome dynamics. Pharmacological agents that upregulate MFF or PEX11β could therefore be of therapeutic value to restore peroxisome dynamics in certain disease conditions.

## MATERIALS AND METHODS

### Plasmids and antibodies

See Table S1 for details of plasmids used in this study, Table S2 for plasmids generated in this study, and Table S3 for details of oligonucleotides used. Site-directed mutagenesis to generate point mutations was performed using the QuikChange Site-Directed Mutagenesis kit (Agilent Technologies). All constructs produced were confirmed by sequencing (Eurofins Genomics). Details on antibodies used in this study can be found in Table S4.

### Cell culture and transfection

MFF-deficient fibroblasts (dMFF; provided by F. S. Alkuraya, King Faisal Specialist Hospital and Research Center, Riyadh, Saudi Arabia; Shamseldin et al., 2012), human control fibroblasts (C109), PEX11β- (dPEX11β) and PEX19-deficient (dPEX19) fibroblasts (provided by H. Waterham, Academic Medical Center, University of Amsterdam, Amsterdam, The Netherlands; Ebberink et al., 2012; Matsuzono et al., 1999), mouse embryonic fibroblasts (including *mff* and *fis1* single and double KO MEFs; provided by D. C. Chan, California Institute of Technology, USA; Losón et al., 2013), and COS-7 cells (African green monkey kidney cells, CRL-1651; ATCC) were cultured in DMEM, high glucose (4.5 g/l) supplemented with 10% FBS, 100 U/ml penicillin and 100 µg/ml streptomycin at 37°C (all from Life Technologies; complete medium) with 5% CO_2_ and 95% humidity. All cells were regularly tested for contamination and mycoplasmas. Informed consent and approval was obtained when the patient fibroblast cell lines were generated (Shamseldin et al., 2012; Matsuzono et al., 1999; Ebberink et al., 2012), in accordance with institutional guidelines and the Dutch Code of Conduct. COS-7 cells were transfected using diethylaminoethyl-dextran (Sigma-Aldrich) (Bonekamp et al., 2010) or Lipofectamine (Invitrogen, Thermo Fisher Scientific). Fibroblasts were transfected by microporation using the Neon Transfection System (Thermo Fisher Scientific) following the manufacturer's protocol. Briefly, cells (seeded 24 h before transfection) were washed once with phosphate-buffered saline (PBS) and trypsinised using TrypLE Express (Thermo Fisher Scientific). Trypsinised cells were resuspended in complete medium, pelleted by centrifugation and washed with PBS. The cells were once again centrifuged and carefully resuspended in 110 µl buffer R. For each condition, 4×10^5^ cells were mixed with the DNA construct (5–10 µg) or with 50–100 nM siRNA (Table S3). Cells were microporated using a 100 µl Neon tip with the following settings: 1400 V, 20 ms, one pulse. Microporated cells were immediately seeded into plates with pre-warmed complete medium without antibiotics and incubated at 37°C with 5% CO_2_ and 95% humidity.

### Analysis of ALS patients with Val241Met genetic variant

The Val241Met genetic variant (p.V241M) has been detected in ALS patients 1–3. The variant has been detected by whole-exome sequencing, while mutations in established ALS genes were excluded. An expansion of the hexanucleotide repeat in C9ORF72 has also been excluded, using fragment analysis and repeat-primed PCR (Hübers et al., 2014). All three patients are part of a German–Swedish ALS whole-exome sequencing project that includes a total of 551 mostly familial ALS index patients. The respective results have been published by Freischmidt et al. (2015) and Müller et al. (2018). The following clinical information is available about the three patients. All patients fulfilled the El Escorial criteria for definitive or probable ALS. Patient 1: the female patient from Finland presented with rapidly progressing dementia with muscle wasting in the limbs and bulbar-innervated muscles. She died in 2003 at age 69 years. Her sister had a similar disease and died several years before (no DNA sample available). Patient 2: this male patient from Germany died from spinal onset ALS at the age of 71 years. No indication of frontotemporal dementia (FTD) co-morbidity was noted. The father of the patient died from ALS at the age of 65 years, no further clinical information is available. Patient 3: this German patient suffered from sporadic ALS and died at the age of 56 years. The diagnosis was neuropathologically confirmed at autopsy. Clinical signs indicated upper motor neuron predominant disease, starting at the upper extremities. The patient did not suffer from FTD co-morbidity. For the genetic analyses of ALS patients, written informed consent was obtained from all individuals. The experiments have been approved by the local ethical committees of the Medical Faculties Ulm (Ulm University) and Mannheim (ethical committee II of the University of Heidelberg). Approval numbers are Nr. 19/12 and 2020-678 N, respectively.

### Immunofluorescence and microscopy

Cells were processed for immunofluorescence 24 h after transfection (if not indicated otherwise). Cells grown on glass coverslips were fixed with 4% paraformaldehyde in PBS, pH 7.4. For detection of PEX11β, cells were permeabilised with 2.5 μg/ml digitonin, as PEX11β is extracted from peroxisome membranes after post-fixation Triton X-100 treatment (Bonekamp et al., 2013a; Schrader et al., 2012). For other detections or differential permeabilisation, cells were either permeabilised with 0.2% Triton X-100 or 2.5 μg/ml digitonin. Cells were incubated with primary and secondary antibodies as described previously (Bonekamp et al., 2013b) (Table S4). Cell imaging was performed using an IX81 microscope (Olympus) equipped with an UPlanSApo 100×/1.40 oil objective (Olympus). Digital images were taken with a CoolSNAP HQ2 CCD camera and adjusted for contrast and brightness using the Olympus Soft Imaging Viewer software and MetaMorph 7 (Molecular Devices).

### Transmission electron microscopy

Sample preparation for transmission electron microscopy was performed as described previously (Costello et al., 2017a). In brief, cells were fixed in 0.5% glutaraldehyde in 0.2 M PIPES buffer (pH 7.2), post-fixed in 1% osmium tetroxide (reduced with 1.5% w/v potassium ferrocyanide) in 0.1 M sodium cacodylate (pH 7.2), followed by dehydration in a graded ethanol series and embedment in Durcupan resin (Sigma-Aldrich, Merck group). Embedded samples were used to prepare 60 nm ultrathin sections, which were collected on 100 mesh copper EM grids, then contrasted with lead citrate and imaged using a JEOL JEM 1400 transmission electron microscope fitted with a digital camera (Gatan, ES1000W).

### Cell lysis and immunoprecipitation

To prepare whole-cell lysates for assaying protein expression, cells were washed in PBS and then lysed in ice-cold lysis buffer [25 mM Tris-HCl, pH 7.5, 150 mM NaCl, 1% Triton X-100, 0.1% SDS and protease inhibitor cocktail (Roche)]. Lysates were incubated on ice for 20 min, and undissolved material was pelleted by centrifugation at 15,000 ***g***. The supernatant was diluted with Laemmli buffer to a concentration of 0.5 mg/ml prior to immunoblotting.

For immunoprecipitation (IP) experiments Myc–PEX11β^WT^, Myc–PEX11β^ΔC5^ or Myc–PEX11β^ΔGly^ were co-expressed with FLAG–FIS1 in COS-7 cells (500,000 cells per IP). After 48 h, cells were incubated with 1 mM 1,8-bis-maleimidodiethyleneglycol (DSP) in PBS for 30 min, followed by quenching with 50 mM Tris-HCl pH 7.5, and were then lysed in 0.5 ml ice-cold IP lysis buffer (25 mM Tris-HCl, pH 7.5, 150 mM NaCl, 1% NP-40, 1 mM PMSF and protease inhibitor cocktail), undissolved material was pelleted by centrifugation at 15,000 ***g***, followed by a second centrifugation step at 100,000 ***g***. IP was performed according to the manufacturer's instructions (ChromoTek). Briefly, clarified lysates were mixed 1:1 with dilution buffer (25 mM Tris-HCl, pH 7.5, 150 mM NaCl, 1 mM PMSF and protease inhibitor cocktail), then mixed with 25 µl Myc-Trap magnetic agarose beads (ChromoTek) and incubated for 2 h at 4°C. Beads were subsequently washed extensively with dilution buffer, and bound proteins were eluted with 50 µl 2× Laemmli buffer. Immunoprecipitates and total lysates were analysed by immunoblotting.

### SDS–PAGE and immunoblotting

Proteins were separated by SDS–PAGE and subsequently transferred to nitrocellulose membranes (Amersham Bioscience) using a semi-dry apparatus (Trans-Blot SD, Bio-Rad). Membranes were blocked in 5% dry milk (Marvel) in Tris-buffered saline with 0.1% Tween-20 (TBS-T) and incubated with primary antibodies (Table S4), followed by incubation with horseradish peroxidase-conjugated secondary antibodies (Table S4) and detection with enhanced chemiluminescence reagents (Amersham Bioscience) using Amersham hyperfilm (GE Healthcare) or the G:Box Chemi (Syngene).

### Data analysis and presentation

Protein sequence alignment was performed using Clustal Omega (1.2.4) or MUSCLE multiple sequence alignment (Madeira et al., 2019). For quantitative analysis of peroxisome morphology, 100–200 cells per coverslip were examined blind and categorised as cells with spherical (0.1–0.3 µm) or elongated [>3 µm in length; rod-shaped, 0.5–2 µm (PEX11β-deficient cells only)] peroxisomes (see Fig. S6 for examples). Usually 2–3 coverslips per preparation were analysed manually, and at least three independent experiments were performed. Overexpressing cells used for quantification showed an intensity level of 4000–5000 units, ensuring that cells with similar fluorescence intensity (expression level) were used for the analysis. Representative images were processed with MetaMorph (Molecular Devices) and ImageJ (NIH) software (Schneider et al., 2012). GraphPad Prism (v8.1.0) was used to perform stastistical analyses and prepare graphs. Significant statistical differences between multiple groups were determined using ordinary one-way ANOVA with Tukey's multiple comparisons (Fig. 3B) or Brown–Forsythe and Welch one-way ANOVA with Dunnett's multiple comparisons where variances were unequal (Figs 1E, 2C, 5D). For multiple variables, two-way ANOVA with Sidak's multiple comparisons was performed (Fig. 4B). *P<0.05, **P<0.01, ***P<0.001. Symbol colour matches the condition from which significant difference is observed. Data are presented as mean±s.d.

## Supplementary Material

Supplementary information
