# Supplementary material for: PEX11β and FIS1 cooperate in peroxisome division independently of mitochondrial fission factor
Source: J Cell Sci. 2022 Jul 8;135(13):jcs259924. doi: 10.1242/jcs.259924 (PMC9377713; doi:10.1242/jcs.259924)
Supplement: Supplementary information [file joces-135-259924-s1.pdf]

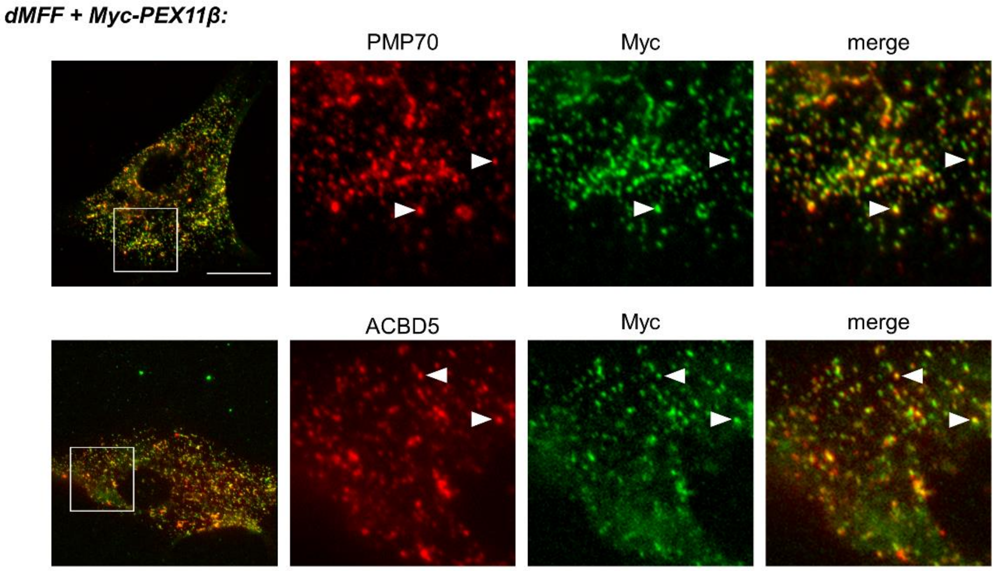

Fig. S1. Localisation of peroxisomal marker proteins at PEX11β-positive peroxisomes. MFF-deficient fibroblasts (dMFF) were transfected with Myc-PEX11β and peroxisomes labelled with anti-Myc and anti-PMP70 or anti-ACBD5 antibodies. Note that the spherical peroxisomes formed by PEX11β-induced division are positive for markers of mature peroxisomes. Bar, 20 µm.

Journal of Cell Science • Supplementary information

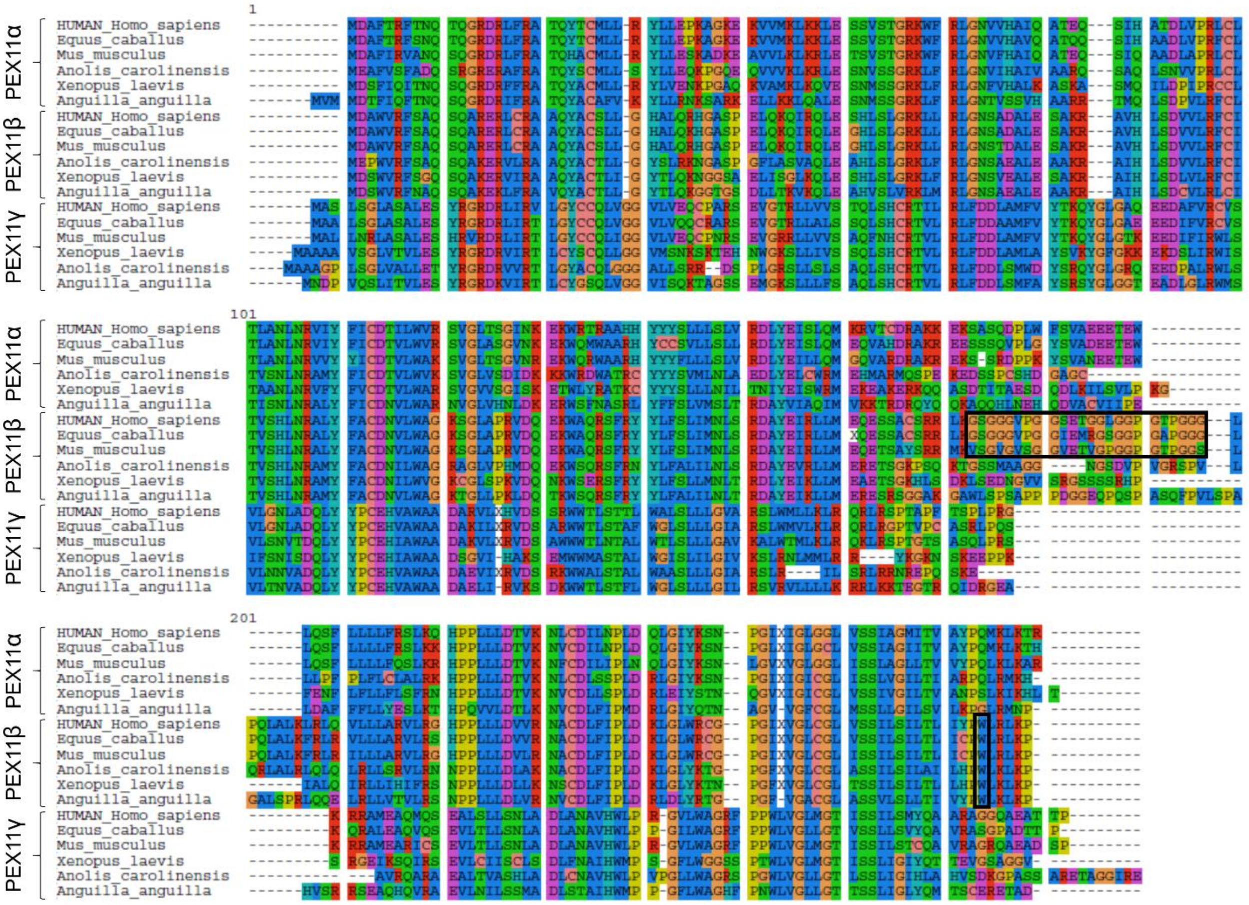

Fig. S2. Alignment of PEX11 protein sequences. Alignment of PEX11 protein sequences, comparing the α, β and γ isoforms of different vertebrate species. The glycine-rich region (only present in mammalian PEX11β) and C-terminal tryptophan residue (only present in PEX11β) are indicated with black boxes.

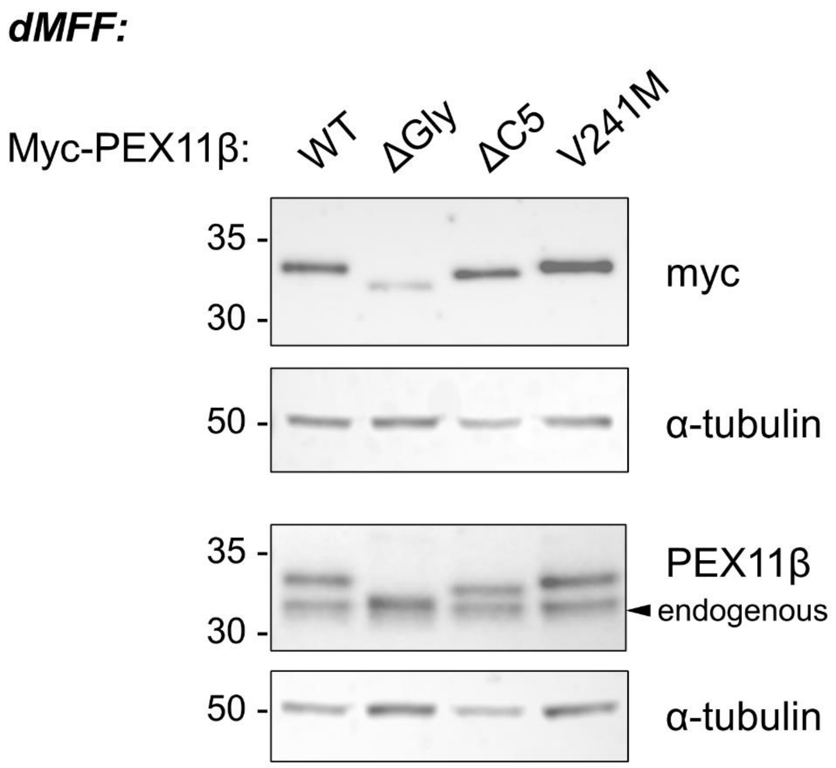

Fig. S3. Expression of PEX11β mutants in dMFF cells. dMFF cells were transfected with Myc-PEX11β^{WT}, Myc-PEX11β$^{\Delta Gly}$, Myc-PEX11β$^{\Delta C5}$, and Myc-PEX11β^{V241M} and processed for immunoblotting after 24 hours using anti-PEX11β and anti-Myc antibodies. Anti-tubulin served as a loading control, immunoblot molecular weight markers shown in kDa. Note that there is no partial degradation of the mutated proteins.

Journal of Cell Science • Supplementary information

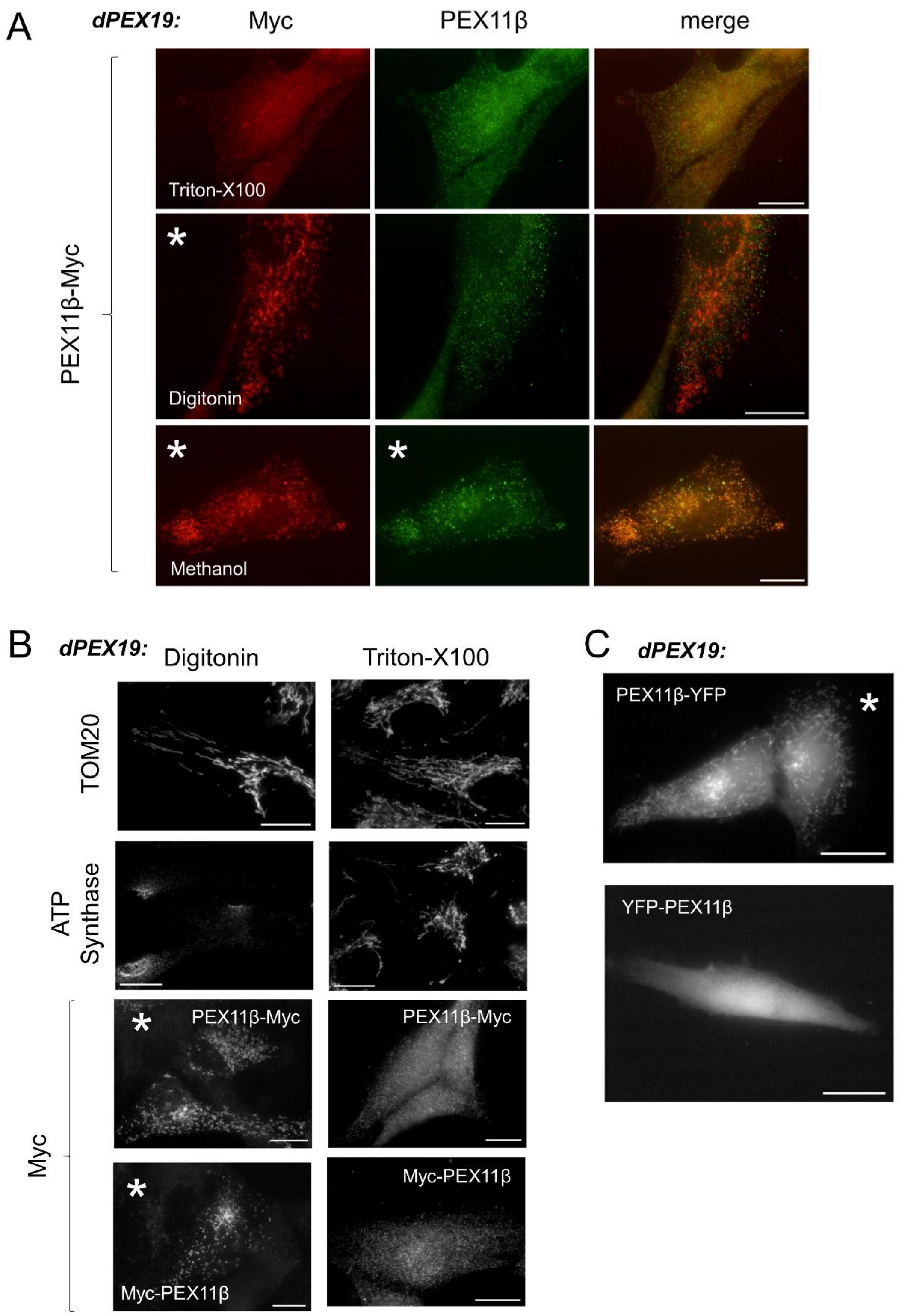

Fig. S4. PEX11β inserts into the mitochondrial membrane with the same topology as in peroxisomes. (A) Representative images of PEX11β detection in dPEX19 cells transfected with PEX11β-Myc and permeabilised with Triton-X100, digitonin or methanol. Fixed cells were labelled with anti-Myc and anti-PEX11β (directed against an

Journal of Cell Science • Supplementary information

epitope within the internal loop of PEX11β) antibodies. Note some background staining of the PEX11β antibody. (B) Representative images of dPEX19 cells transfected with Myc-PEX11β or PEX11β-Myc and permeabilized with Triton-X100 or digitonin. Fixed cells were labelled with anti-Myc. Labelling with anti-TOM20 (directed to cytoplasmic epitope) and anti-ATP synthase (directed to mitochondrial matrix epitope) acted as permeabilisation controls. In (A, B), note that postfixation Triton-X100 treatment extracts PEX11β from membranes, as previouslyreported (Schrader et al., 2012). Mitochondrial localisation of Myc-PEX11β/PEX11β-Myc is detected by anti-Myc antibodies in cells permeabilised with digitonin, which only permeabilises the plasma membrane, suggesting both termini are exposed to the cytosol. However, anti-PEX11β antibodies do not detect PEX11β-Myc at mitochondria under these conditions, suggesting the epitope recognised by this antibody is inside the mitochondria, and is only accessible after methanol treatment, which permeabilises all membranes. This is consistent with the PO topology of PEX11β shown in Fig. 2A. (C) Representative images of dPEX19 cells transfected with PEX11β-YFP or YFP-PEX11β. Note that tagging of the N-terminus inhibits targeting of PEX11β to mitochondria in dPEX19 cells. * indicates PEX11β detection at mitochondria. Bars, 20 μm.

Journal of Cell Science • Supplementary information

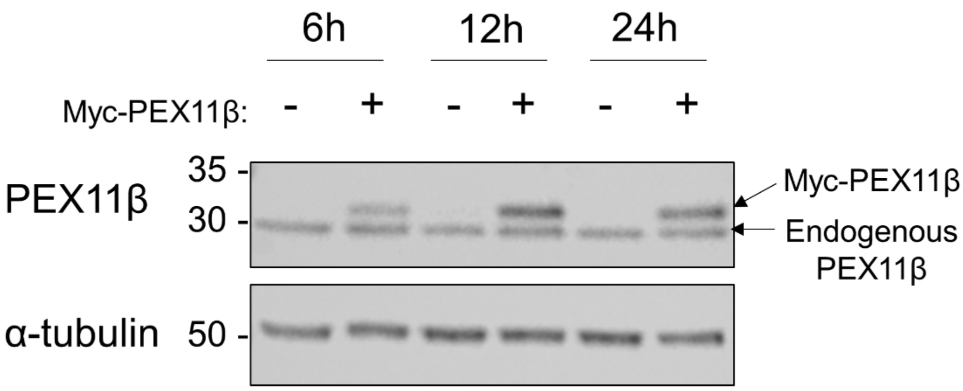

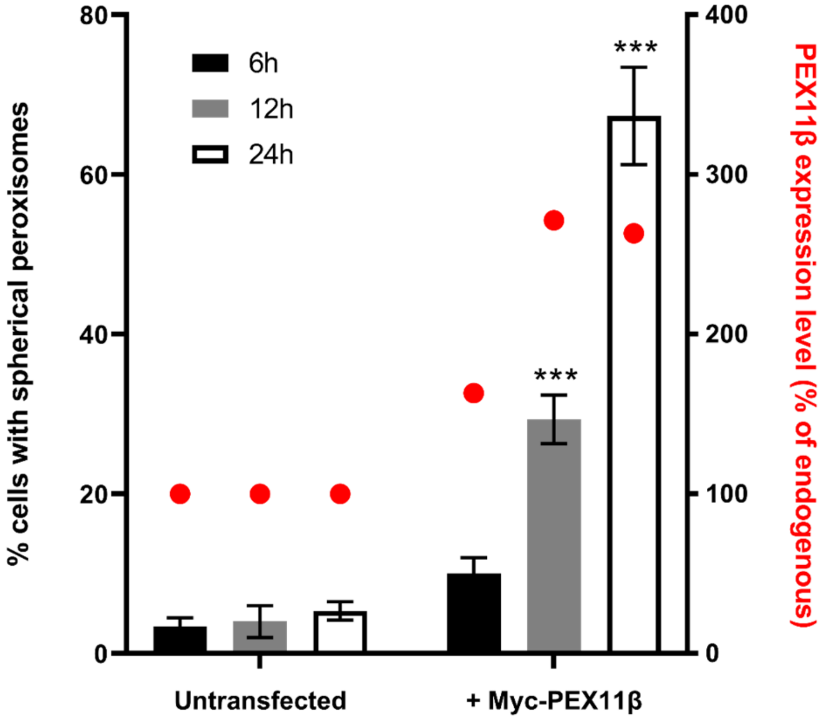

Fig. S5. Expression level of PEX11β and peroxisome division in dMFF cells after different time points. dMFF cells were transfected with Myc-PEX11b and processed for immunofluorescence and immunoblotting after 6, 12 and 24 hours. For immunofluorescence, cells were labelled with anti-Myc and anti-PEX14 antibodies and peroxisome morphology quantified as previously described. Corresponding protein samples were processed for immunoblotting using anti-PEX11β antibodies. Anti-tubulin served as a loading control, immunoblot molecular weight markers shown in kDa. PEX11β expression was quantified using ImageJ and presented as a ratio of total PEX11β to endogenous PEX11β signal in each sample. Note that an approx. 2.5 fold increase in PEX11β is sufficient to induce peroxisome division in dMFF cells. Data are presented as mean ± SD. *** $P < 0.001$.

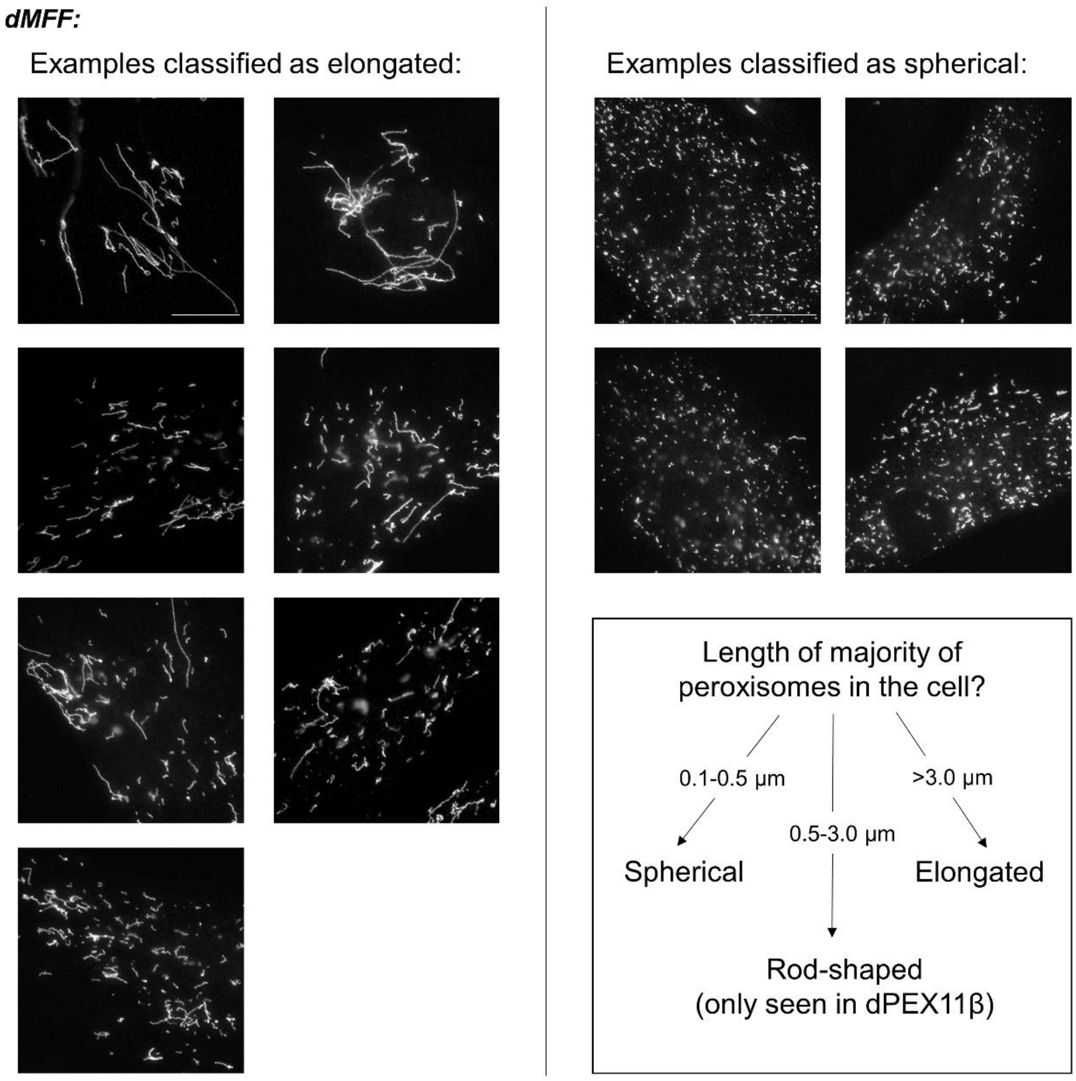

Fig. S6. Examples of cells with an elongated or spherical peroxisome phenotype used for binary quantification.

Please note that MFF-deficient cells contain mainly highly elongated peroxisomes, whereas cells with spherical peroxisomes are rarely observed. Elongation may vary (see left column). Intermediate phenotypes of peroxisome division (e.g. a beads-on-a string phenotype) are more frequent early after PEX11β expression (Passmore et al., 2020), and are rare after 24 hours of expression.

Table S1. Plasmids used in this study.

Plasmid	Source	Vector
5xMyc-MFF	Kindly provided by A. van der Bliek, UCLA, Los Angeles, USA (Gandre-Babbe et al., 2008)	pCS2+MT
3xFLAG-FIS1	Kindly provided by N. Ishihara, Kurume University, Kurume, Japan (Onoue et al., 2013)	p3xFLAG-CMV-10
DRP1-GFP	Kindly provided by M. McNiven, Mayo Clinic, Rochester, USA (Pitts et al., 1999)	pEGFP-C1
Myc-PEX11α	Delille et al., 2010	pCMV-tag3A
PEX11α-3xFLAG	Kindly provided by C. Brocard, University of Vienna, Vienna, Austria (Koch et al., 2010)	pReceiverM14
Myc-PEX11β	Delille et al., 2010	pCMV-tag3A
PEX11β-Myc	Schrader et al., 1998	pcDNA3
Myc-PEX11γ	Schrader et al., 2012	pCMV-tag3A
PEX11β-Myc W4A	Williams et al., 2015	pcDNA3
PEX11β-Myc L48A	Williams et al., 2015	pcDNA3
PEX11β-Myc W4A/L48A	Williams et al., 2015	pcDNA3
PEX11β-Myc ΔN40	Bonekamp et al., 2013	pcDNA3
PEX11β-Myc A21P	Bonekamp et al., 2013	pcDNA3
Myc-PEX11β ΔGly	Bonekamp et al., 2013	pCMV-Tag3A
Myc-PEX11β ΔC5	Delille et al., 2010	pCMV-Tag3A

Table S2. Plasmids generated in this study.

Plasmid	Template	Primers	Enzymes	Vector
PEX11β-Myc W4E	PEX11β-Myc	PEX11β_W4E_F, PEX11β_W4E_R	-	pcDNA3
PEX11β-Myc L59P	PEX11β-Myc	PEX11β_L59_F, PEX11β_L59_R	-	pcDNA3
PEX11β-Myc D66P	PEX11β-Myc	PEX11β_D66_F, PEX11β_D66_R	-	pcDNA3
PEX11β-Myc L59P/D66P	PEX11β-Myc L59P	PEX11β_D66_F, PEX11β_D66_R	-	pcDNA3
PEX11β-Myc ΔN12	PEX11β-Myc	PEX11β_-ATG_F, PEX11β_-ATG_R; PEX11β_+ATG_F, PEX11β_+ATG_R	-	pcDNA3
Myc-PEX11β N only	Myc-PEX11β, ACBD5.2 (pCMV-Tag3A)	PEX11βup, PEX11β_ACBD5_F PEX11β_ACBD5_R, ACBD5-EcoRI_R	BamHI, EcoRI	pCMV-Tag3A
Myc-PEX11β αC7	Myc-PEX11β	PEX11β_αC7_F, PEX11β_αC7_R	-	pCMV-Tag3A
Myc-PEX11β W254A	Myc-PEX11β	PEX11β_W254A_F, PEX11β_W254A_R	-	pCMV-Tag3A
Myc-PEX11β V241M	Myc-PEX11β	PEX11β_V241M_F, PEX11β_V241M_R	-	pCMV-Tag3A

Table S3. Oligonucleotides used in this study.

Primers:

Name	Sequence (5' to 3')
PEX11β_W4E_F	AAGCTTGGTACCATGGACGCCGAGGTCCGCTTCAGTGCTCAGAGC
PEX11β_W4E_R	GCTCTGAGCACTGAAGCGGACCTCGGCGTCCATGGTACCAAGCTT
PEX11β_L59_F	CTTGGAAGAAAGCTTCCACGCCTGGGTAACTCAG
PEX11β_L59_R	CTGAGTTACCCAGGCGTGGAAGCTTTCTTCCAAG
PEX11β_D66_F	CTGGGTAACTCAGCACCTGCCCTTGAGTCAGC
PEX11β_D66_R	GCTGACTCAAGGGCAGGTGCTGAGTTACCCAG
PEX11β_-ATG_F	ACCCAAGCTTGGTACCACGGGACGCCTGGGTCCG
PEX11β_-ATG_R	CGGACCCAGGCGTCCCGTGGTACCAAGCTTGGGT
PEX11β_+ATG_F	TCCGCTTCAGTGCTCAGAGCCATGGCCCGGGAGCGGCTGTGTAGG
PEX11β_+ATG_R	CCTACACAGCCGCTCCCGGGCCATGGCTCTGAGCACTGAAGCGGA
PEX11βup	TTGGATCCTATGGACGCCTGGGTCCGCTTC
P11b_ACBD5_R	CAAAGGGCCACCATCGATTGAGGTGACTAACAGTG
P11b_ACBD5_R	CACCTCAATCGATGGTGGCCCTTTGAGATGTCC
ACBD5-EcoRI_R	CCCGAATTCCTTCAATTTAGTTTTCTTCTCCTTCTTTG
PEX11β_αC7_F	ATTCTCACCCTAATCTATCCCCAGATGAAGCTGAAGACCCGTTGAGAATTCGATATCAAGCTT
PEX11β_αC7_R	AAGCTTGATATCGAATTCTCAACGGGTCTTCAGCTTCATCTGGGGATAGATTAGGGTGAGAAT
PEX11β_W254A_F	ATTCTCACCCTAATCTATCCCGCGCTACGACTCAAGCCCTGAGAA
PEX11β_W254A_R	TTCTCAGGGCTTGAGTCGTAGCGCGGGATAGATTAGGGTGAGAAT
PEX11β_V241M_F	GGGCTTTGTGGCCTCATGTCCTCCATCCTGTCT
PEX11β_V241M_R	AGACAGGATGGAGGACATGAGGCCACAAAGCCC

siRNA:

Name	Sequence (5' to 3')	Source
siDRP1	UCCGUGAUGAGUAUGCUUU	siMAX, Eurofins
siFIS1	GGAAUACGAGAAGGCCUUA	Thermofischer, ID: 23959
siControl	UAAGGCUAUGAAGAGAUAC<br>AUGUAUUGGCCUGUAUUAG<br>AUGAACGUGAAUUGCUCAA<br>UGGUUUACAUGUCGACUAA	siGENOME Non-targeting siRNA pool, Dharmacon

Table S4. Primary and secondary antibodies used in this study.

Antibody	Type	Dilution		Source
		IMF	WB	
DRP1	mc ms	-	1:750	BD Transduction 611112
FIS1	pc rb	-	1:1,000	Proteintech 10956-1-AP
FLAG	mc ms	1:500	-	Sigma F3165
FLAG	pc rb	1:750	1:1,000	Sigma F7425
GAPDH	pc rb	-	1:20,000	ProSci 3783
MFF	pc rb	-	1:2,000	A. M. van der Bliek, UCLA, USA
Myc	mc ms	1:200	-	Santa Cruz Biotechnology, Inc 9E10
Myc	mc rb	1:200	1:2,000	Abcam ab9106
PEX11β	pc rb	1:200	-	Abcam ab74507
PEX11β	mc rb	-	1:1,000	Abcam ab181066
PEX14	pc rb	1:1,400	-	D. Crane, Griffith University, Brisbane, Australia
PMP70	mc ms	1:50	-	Sigma SAB4200181
ACBD5	pc rb	1:100	-	Cambridge Bioscience HPA012145
TOM20	mc ms	1:200	-	BD Transduction 612278
α-tubulin	mc ms	-	1:10,000	Sigma T9026
Alexa Fluor 488 IgG	dk anti-ms	1:400	-	Molecular Probes
Alexa Fluor 594 IgG	dk anti-rb	1:1,000	-	Molecular Probes
HRP IgG	gt anti-rb	-	1:10,000	Bio-Rad Laboratories 170-6515
HRP IgG	gt anti-ms	-	1:10,000	Bio-Rad Laboratories 170-6516

Abbreviations: mc, monoclonal; pc, polyclonal; ms, mouse; rb, rabbit; dk, donkey; gt, goat; HRP, horseradish peroxidase, IMF, immunofluorescence; WB, western blot.

References

Bonekamp, N.A., S. Grille, M.J. Cardoso, M. Almeida, M. Aroso, S. Gomes, A.C. Magalhaes, D. Ribeiro, M. Islinger, and M. Schrader. 2013. Self-interaction of human Pex11pbeta during peroxisomal growth and division. PLoS One. 8:e53424. doi:10.1371/journal.pone.0053424

Delille, H.K., B. Agricola, S.C. Guimaraes, H. Borta, G.H. Lüers, M. Fransen, and M. Schrader. 2010. Pex11pbeta-mediated growth and division of mammalian peroxisomes follows a maturation pathway. J. Cell Sci. 123:2750–2762. doi:10.1242/jcs.062109

Gandre-Babbe, S., and A.M. van der Bliek. 2008. The novel tail-anchored membrane protein Mff controls mitochondrial and peroxisomal fission in mammalian cells. Mol. Biol. Cell. 19:2402–2412. doi:10.1091/mbc.E07

Koch, J., K. Pranjic, A. Huber, A. Ellinger, A. Hartig, F. Kragler, and C. Brocard. 2010. PEX11 family members are membrane elongation factors that coordinate peroxisome proliferation and maintenance. J. Cell Sci. 123:3389–3400. doi:10.1242/jcs.064907

Onoue, K., A. Jofuku, R. Ban-Ishihara, T. Ishihara, M. Maeda, T. Koshiba, T. Itoh, M. Fukuda, H. Otera, T. Oka, H. Takano, N. Mizushima, K. Mihara, and N. Ishihara. 2013. Fis1 acts as a mitochondrial recruitment factor for TBC1D15 that is involved in regulation of mitochondrial morphology. J. Cell Sci. 126:176–185. doi:10.1242/jcs.111211

Passmore, J.B., R.E. Carmichael, T.A. Schrader, L.F. Godinho, S. Ferdinandusse, C. Lismont, Y. Wang, C. Hacker, M. Islinger, M. Fransen, D.M. Richards, P. Freisinger, and M. Schrader. 2020. Mitochondrial fission factor (MFF) is a critical regulator of peroxisome maturation. Biochim. Biophys. Acta. - Mol. Cell Res. 1867(7):118709. doi: 10.1016/j.bbamcr.2020.118709

Pitts, K.R., Y. Yoon, E.W. Krueger, and M.A. McNiven. 1999. The dynamin-like protein DLP1 is essential for normal distribution and morphology of the endoplasmic reticulum and mitochondria in mammalian cells. Mol. Biol. Cell. 10:4403–4417. doi:10.1091/mbc.10.12.4403

Journal of Cell Science • Supplementary information

Schrader, M., M. Almeida, and S. Grille. 2012. Postfixation detergent treatment liberates the membrane modelling protein Pex11beta from peroxisomal membranes. Histochem. Cell Biol. 138:541–547.

Schrader, M., B.E. Reuber, J.C. Morrell, G. Jimenez-Sanchez, C. Obie, T.A. Stroh, D. Valle, T.A. Schroer, and S.J. Gould. 1998. Expression of PEX11beta mediates peroxisome proliferation in the absence of extracellular stimuli. J. Biol. Chem. 273:29607–29614.

Williams, C., L. Opalinski, C. Landgraf, J. Costello, M. Schrader, A.M. Krikken, K. Knoops, A.M. Kram, R. Volkmer, and I.J. van der Klei. 2015. The membrane remodeling protein Pex11p activates the GTPase Dnm1p during peroxisomal fission. Proc. Natl. Acad. Sci. U. S. A. 112:6377–6382. doi:10.1073/pnas.1418736112

Journal of Cell Science • Supplementary information
